# Identifying presence or absence of grizzly and polar bear cubs from the movements of adult females with machine learning

**DOI:** 10.1186/s40462-025-00577-y

**Published:** 2025-07-04

**Authors:** Erik M. Andersen, Justin G. Clapp, Milan A. Vinks, Todd C. Atwood, Daniel D. Bjornlie, Cecily M. Costello, David D. Gustine, Mark A. Haroldson, Lori L. Roberts, Karyn D. Rode, Frank T. van Manen, Ryan R. Wilson

**Affiliations:** 1 U.S. Fish and Wildlife Service, Marine Mammals Management, 1011 E Tudor Road, Anchorage, AK 99503 USA; 2 Wyoming Game and Fish Department, Large Carnivore Section, 260 Buena Vista, Lander, WY 82520 USA; 3 Montana Fish, Wildlife and Parks, 490 N Meridian Rd, Kalispell, MT 59901 USA; 4 Montana Cooperative Wildlife Research Unit, Wildlife Biology Program, University of Montana, 205 Natural Sciences Building, Missoula, MT 59812 USA; 5 Alaska Science Center, U.S. Geological Survey, 4210 University Drive, Anchorage, AK 99508 USA; 6 Biological Resources Division-Wildlife Conservation, National Park Service, Natural Resource Stewardship and Science, Anchorage, AK 99502 USA; 7 Northern Rocky Mountain Science Center, Interagency Grizzly Bear Study Team, U.S. Geological Survey, 2327 University Way, Suite 2, Bozeman, MT 59715 USA

**Keywords:** Support vector machine, Brown bear, Ursid, Litter survival, Polar bear, Reproductive class, Reproductive success, Learning algorithm, Movement metrics, Clustering

## Abstract

**Background:**

Information on reproductive success is crucial to understanding population dynamics but can be difficult to obtain, particularly for species that birth while denning. For grizzly (*Ursus arctos*) and polar bears (*U. maritimus*), den visits are impractical because of safety and logistical considerations. Reproduction is typically documented through direct observation, which can be difficult, costly, and often occurs long after den departure. Reproduction could be documented remotely, however, from post-denning movement data if discernable differences exist between females with and without cubs.

**Methods:**

We trained support vector machines (SVMs) with eight variables derived from telemetry data of female grizzly (2000–2022) and polar bears (1985–2016) with or without cubs during seven periods with lengths ranging from 5 to 60 days starting at den departure. We assessed SVM classification accuracy by withholding two samples (one cub-present, one cub-absent), training SVMs with the remaining data, predicting classification of the withheld samples, and repeating this process for each sample combination. Additionally, we evaluated how classification accuracy for grizzly bears was influenced by sample size, length of the post-departure period, and frequency of standardized location estimates.

**Results:**

Accuracy of predicting cub presence or absence was 87% for grizzly bears with only 5 days of post-departure data and increased to a maximum of 92% with 20 days of data. For polar bears, accuracy was 86% at 5 days post-departure and increased to a maximum of 93% at 50 days. Classification accuracy for grizzly bears increased from 76 to 90% when sample size increased from 10 to 30 bears while holding period length constant (30 days) but did not increase at larger sample sizes. When sample size was held constant, increasing the length of the post-departure period did not affect classification accuracy markedly.

**Conclusion:**

Presence or absence of grizzly and polar bear cubs can be identified with high accuracy even when SVM models are trained with limited data. Detecting cub presence or absence remotely could improve estimates of reproductive success and litter survival, enhancing our understanding of factors affecting cub recruitment.

**Supplementary Information:**

The online version contains supplementary material available at 10.1186/s40462-025-00577-y.

## Background

Information on age-specific reproduction is crucial to understanding population dynamics of wildlife [1]. Documenting the timing, duration, and frequency of reproductive events is a common aspect of population monitoring protocols, and such data are often incorporated into demographic models used to estimate population vital rates, abundance, and trend. However, for some species, reproduction is difficult to document because of low densities, cryptic behaviors, inaccessible habitats, human safety concerns, or risk of disturbance. Among large mammals, the use of very high frequency (VHF) or global positioning system (GPS) tracking devices coupled with aerial or ground observations has enhanced the ability to document spatiotemporal aspects of reproduction and has facilitated the estimation of reproductive parameters such as litter size. The use of vaginal implant transmitters, which transmit a VHF or satellite signal when expelled at parturition, has improved monitoring of reproduction and neonates in ungulate species [2, 3].

One group of animals for which documentation of reproduction is challenged through behavior are those that give birth while denning, including several species of bears within the subfamily Ursinae. In most parts of their range, American black bears (*Ursus americanus*) and grizzly bears (*U. arctos*) overwinter in dens, entering hibernation as an adaptation to coping with seasonal lack of food and adverse winter weather conditions [4]. While denning, bears become inactive, do not feed or drink, and exhibit a variety of physiological changes in response to fasting. The denning period is particularly critical for pregnant females because denning provides a protected place to give birth and undergo lactation until neonates are large enough to survive conditions outside the den [5, 6]. Parturient American black bears and grizzly bears typically enter dens in autumn, give birth during December–February, and emerge from dens between March and May. Polar bears (*U. maritimus*), however, have access to prey throughout winter, so only pregnant females den for extended periods during winter and typically give birth in December or January and emerge from dens in March or April [7, 8].

Reproductive success of denning female American black bears can be determined by direct visual observation within the den or by tranquilizing the female, which are effective methods to document reproduction, litter size, sex ratio, and offspring condition. However, these approaches are impractical for polar and grizzly bears which are more reactive and dangerous to humans during denning. As a result, reproduction by polar bears and grizzly bears can typically only be determined via visual observations of bears after den emergence in spring. However, monitoring den sites to allow visual observation at den emergence is logistically difficult, particularly because den sites often occur in remote areas. Further, complete litter loss may go undetected when females are observed days or weeks post-den emergence.

The ability to reliably estimate whether individual radio-marked female bears emerge from their dens with offspring can contribute to demographic assessments by providing input on natality, which can facilitate more accurate estimates of litter survival when bears are observed subsequently [9]. Such data are particularly important because natality and survival of young are often the first vital rates to be affected by environmental change [10], making reproduction particularly important to monitor. For example, changes in litter production or prevalence of whole litter loss can provide crucial insights into how population demographics may be affected by intrinsic factors such as density dependence [11, 12] or extrinsic factors such as climate change [13]. Studies examining factors that affect litter survival of bears usually require visual observations of offspring presence or absence (e.g., [7, 8]; however, this often leads to small sample sizes given the constraints of surveying remote areas where maternal females occur.

Movement and space use data from adult female bears could signal presence or absence of cubs-of-the-year (henceforth, ‘cubs’) if discernable differences in movement exist between these two groups. For example, female grizzly bears with cubs typically showed restricted movements and remained at higher elevations in the first few weeks after den departure relative to bears without cubs [14, 15]. Low mobility of cubs likely contributed to these movement patterns, but the reduced movement rates and spatial separation of females with cubs from other bears may have also served to protect vulnerable cubs from predation [12, 14]. Similarly, polar bears observed with young cubs after den departure moved slower than solitary females [16, 17] or females with yearlings [17] and telemetry data suggested that movement was slowest immediately after den departure [18]. Female polar bears with cubs exhibited lower movement rates than solitary adult females and other reproductive classes of bears until about 1–2 months after departing the den site [17]. Thus, there is evidence that movement patterns differ between females with and without cubs that could be used to classify reproductive status. However, unlike species (e.g., ungulates) for which parturition can be identified by abrupt and sustained decreases in movement rates, parturition for bears occurs inside dens when animals are not moving, and post-den departure movements lack break points that have been used successfully to identify neonate birth and loss in other species [e.g., 19].

Classification methods that separate data according to shared features are regularly used to answer ecological questions [20]. These methods include unsupervised classification models to predict unknown states (e.g., state-space models), or supervised classification into known states based on observations from training data (e.g., support vector machines [SVM]). Also referred to as maximum margin classifiers, SVMs are nonlinear, machine-learning algorithms that use features of training data to optimize the separation of observations into classes [21]. SVMs accomplish this separation by identifying the maximum-margin hyperplane, which is defined by maximizing the distance between observations that lie closest to the margin separating the groups (i.e., the support vectors) and the hyperplane itself. Kernel functions are used to map input data onto high-dimensional feature spaces where complex ecological patterns can be more simply represented and separated [22]. Once separation is defined via training data, predictions can be made on new data based on their position relative to the support vectors. SVMs are unaffected by autocorrelated observations, do not require assumptions about the distribution of observations, and provide accurate classification even with limited training data [23–25]. Consequently, SVMs are suitable for addressing a wide array of biological questions [26], and have been used to estimate ecological niches [23], identify species presence or absence [27], classify individuals based on diet [24], estimate ranges of poorly sampled species [28], and infer behavioral states based on location and movement data [20].

We applied SVMs to movement metrics derived from satellite radio collar location data of adult female grizzly and polar bears—two species that differ markedly in habitat requirements and movement dynamics—to evaluate the potential for predicting cub presence or absence at den departure. We further explore how sample size and length of period since den departure affect the accuracy of SVM classifications. The ability to detect cub presence or absence remotely could be important for many research and management applications, improving estimates of reproductive success and litter survival and enhancing our understanding of factors affecting cub recruitment.

## Methods

### Data collection

We obtained GPS location data from reproductive-age (≥ 4 years) female grizzly bears captured using culvert traps or Aldrich leg-hold snares [29] and instrumented with GPS collars (Telonics, Inc., Mesa, Arizona) from two study areas: the Greater Yellowstone Ecosystem in Wyoming, Montana, and Idaho, USA, during 2000–2021, and the Northern Continental Divide Ecosystem in Montana during 2003–2022. Acquisition rates for GPS collars varied from 13 to 360 min. We conducted telemetry flights between early-April and late-May to ascertain the reproductive status of each female and document age of any observed offspring (i.e., cubs, yearlings, or 2-year-olds) and litter size. For females that were not observed during earlier flights because of poor observability (e.g., dense vegetation), we used data from later flights or ground observations throughout the active season (non-denning period) to monitor offspring survival or to fill in missing data.

We obtained location data from reproductive-age (≥ 4 years) female polar bears instrumented with satellite radio collars (Telonics, Inc., Mesa, Arizona) that were captured near the northern and northwestern coasts of Alaska, USA, between mid-March and early-May. Collars were deployed on bears located from a helicopter and immobilized with standard techniques [30] in the southern Beaufort Sea during 1985–2016 and in the adjacent Chukchi Sea during 2008–2017 [31, 32]. Radio collars recorded Argos [33] or GPS location data at intervals ranging from 1 h to 5 days. Observations of previously collared bears occurred during capture efforts, and the presence and age of dependent young was recorded.

### Data processing

For grizzly bears, we removed GPS location records with horizontal errors > 125 m (both populations) or positional dilution of precision values > 10 (Greater Yellowstone Ecosystem only; [34]). For both species, we removed points we considered to be location errors because they were associated with travel speeds exceeding 25 km/hour, which is an implausible sustained speed for these species. For our analyses, we used time series of location data from adult females for a given year beginning at den site departure and ending 60 days after departure (henceforth, ‘bear-years’). We estimated den departure using criteria specific to the movements of each species and the available data. For grizzly bears, we estimated den departure as movement > 250 m from the den site without return during the following 10 location records, which reduced the potential for misclassification of departure due to location error. For polar bear dens on land, we estimated den departure as movement > 3 km from the den site without return during the following 10 records. We used 3 km as the threshold distance because it is twice the estimated error of the lowest quality Argos data we considered (class 1; [33]). For polar bear dens on sea ice, where location data can be confounded by ice movement, we used departure date estimates based on temperature data recorded by collar sensors [8, 35].

We considered only those bear-years for which the adult female was known to be with or without cubs. For both species, the cub-present sample was defined as those females observed with cubs at any time after den departure, because cub presence, even weeks after departure, indicated that cubs were present since den departure. Time series for female grizzly bears with cubs were truncated at the last observation with cubs because the litter could have been lost subsequently. For polar bears, however, observations often occurred soon after den departure. Consequently, to maintain adequate sample sizes, we assumed that cubs survived and remained with the mother after observation. For grizzly bears, the cub-absent sample was restricted to only those females that were known to be without offspring upon entering the den in the fall, were observed without cubs post-denning, and were classified as having not undergone parturition based on activity data [36, 37]. Unlike grizzly bears, only parturient polar bears den for extended periods [7, 8], so a direct analog to grizzly bears (i.e., a female that denned but was not parturient) did not exist. Therefore, our sample of females without cubs included adult females that did not den, and therefore, were known not to have cubs; these adult females may have been solitary or associated with yearlings or two-year-olds. To create a time series for these non-denning bears, we selected ‘den departure’ dates randomly from the empirical distribution of observed den departure dates of females that were known to have denned.

To integrate location data obtained from Argos and GPS while accounting for location error, differences in acquisition rates, and missing data, we used continuous-time correlated random walk models (CRAWL; [38, 39]) to predict maximum likelihood locations at standardized 24-hour intervals for both species. Additionally, we predicted locations at 6-hour intervals (the maximum relocation interval for collared bears in our dataset) for grizzly bears to determine whether capturing movements at finer scales would improve our ability to identify cub presence or absence. We assigned GPS location data an accuracy of 30 m [40] and Argos location classes 0, 1, 2, 3, A, B, and Z accuracies of 6800, 2500, 1000, 400, 4100, 7600, and 4700 m, respectively [41].

### Support vector machine models

**Fig. 1:**
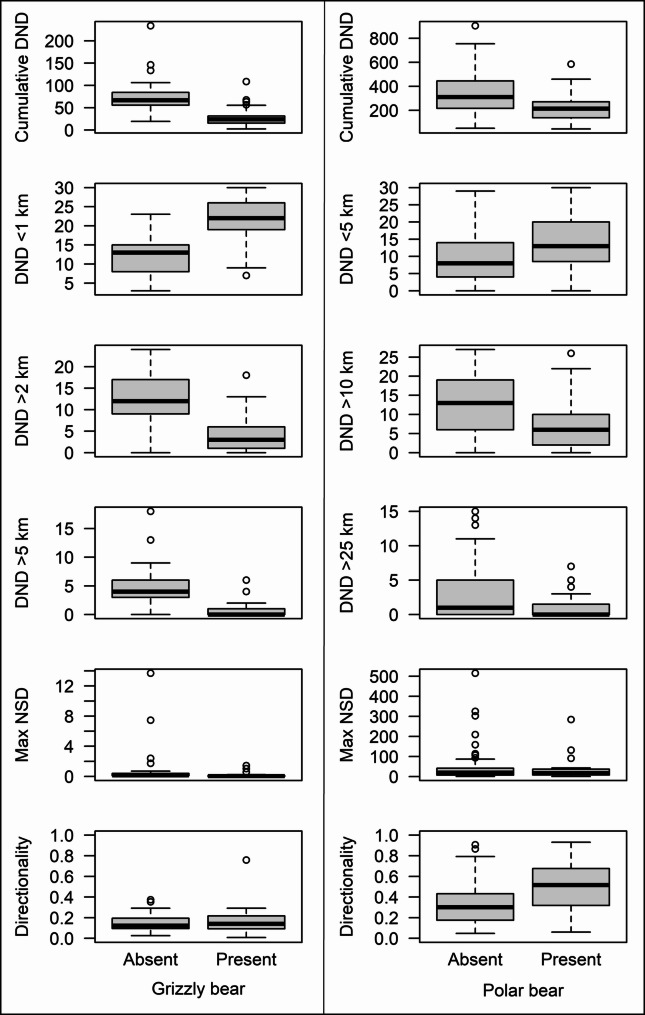
Distributions of six movement metrics of adult female grizzly bears (left) and polar bears (right) during the first 30 days post-den departure used to identify cub presence or absence with support vector machines: cumulative daily net displacement (DND) in km, days with DND < 1 km for grizzly bears or < 5 km for polar bears, days with DND > 2 km for grizzly bears or > 10 km for polar bears, days with DND > 5 km for grizzly bears or > 25 km for polar bears, maximum net-squared displacement (NSD) in gigameters, and mean strength of directionality expressed from 0 to 1. Gray rectangles depict the extent between the lower and upper quartiles with medians indicated by bold black lines; whiskers denote 1.5 times the inter-quartile range above and below the quartiles with outlying points depicted as open circles. Means and standard errors for these metrics during other periods are provided in Supplementary Table S1

We classified presence or absence of cubs from the post-den departure movements of adult females with C-classification SVM models, implemented in the e1071 package for R 4.2.3 [42, 43]. SVM variables included population (grizzly bears) or subpopulation (polar bears), den departure date (Julian day) and 6 variables calculated using the maximum likelihood location estimates from CRAWL models (standardized to 24-hour intervals for both species and 6-hour intervals for grizzly bears) at seven periods of increasing duration starting with den departure (5-, 10-, 20-, 30-, 40-, 50-, and 60-days post-departure). Three movement variables were the same for both species: cumulative daily net displacement (cumulative distance between daily locations in km); maximum net-squared displacement (square of the maximum observed Euclidean distance from the starting location in km); and mean resultant length (a measure of tortuosity that expresses the strength of directionality on a scale from 0 to 1 with smaller values representing greater dispersion of directionality; calculated with the function ‘circ.disp’ from the R package CircStats [44]). The remaining three movement variables differed in spatial scale due to inherent differences in daily movements between the two species; for grizzly bears, these were number of days with daily net displacement < 1 km, > 2 km, and > 5 km, and for polar bears, these were the number of days with daily net displacement < 5 km, > 10 km, and > 25 km. Because some collar data for polar bears included infrequent relocation intervals and gaps in data, we used only those bear-years in each period for which location data existed on at least 75% of the days in each period. We standardized all values to *Z*-scores (mean = 0, SD = 1). Model variable means and standard errors by species and cub presence are provided in Supplementary Table S1 and examples of distributions of the six movement metrics at 30-days post-den departure are depicted in Fig. 1.

We fit separate SVMs for each period with variables from shorter periods included in models for longer periods (e.g., models for the 20-day period included variables for 5-, 10-, and 20-days post-departure). Sample sizes for females with and without cubs were typically asymmetric (Table 1), so we used class weighting to avoid the potential for larger classes to exert disproportionate influence on classification. Because the performance of SVMs can be affected by the choice of model hyperparameters such as the kernel function and penalization parameter (i.e., the ‘cost’ of constraint violations for misclassifications due to non-separable points), model optimization can be achieved through a grid search in which all combinations of hyperparameters across a predetermined range are tested to identify the combination that results in the highest classification accuracy, a process known as ‘model tuning’ [45]. We used the function ‘tune’ (e1071 package; [42]) to identify the optimal kernel function and cost for each temporal scale and subset of data (Table 1). We validated classification accuracy with a leave-two-out cross-validation process that included: (1) withholding two samples (one cub-present and one cub-absent) for testing, (2) training tuned SVMs with the remaining data, (3) predicting the classification of the two test samples, and then (4) repeating this process for each possible combination of testing and training samples. Consequently, models were tested only with data not used for training. We present overall accuracy and the proportion of samples with cubs present and absent that were classified correctly (sensitivity and specificity, respectively).

**Table 1:** Characteristics and validation of tuned support vector machine classification of cub presence or absence based on movement metrics of adult female grizzly bears and polar bears calculated from standardized location estimates at 6- and 24-hour intervals with continuous-time correlated random walk models during seven periods (in days) following den departure (Period). For each period, number of cub-present and cub-absent samples (*n*; representing all available data), sensitivity (Sen; proportion of samples with cubs present that were classified correctly), specificity (Spec; proportion of samples with cubs absent that were classified correctly), and classification accuracy (Acc; proportion of all samples that were classified correctly) were estimated with a leave-two-out cross-validation process that included: (1) withholding two samples (one cub-present and one cub-absent) for testing, (2) training tuned support vector machines characterized by best-performing function kernels (Kernel) and penalizing parameters (Cost) with the remaining data, (3) predicting the classification of the two test samples, and then (4) repeating this process for each possible combination of testing and training samples

Species	Interval	Period	*n*_present_	*n*_absent_	Kernel	Cost	Sen	Spec	Acc
Grizzly bear	24	5	57	25	radial	1	0.89	0.84	0.87
		10	55	25	radial	0.1	0.86	0.80	0.83
		20	52	25	linear	0.1	0.92	0.92	0.92
		30	42	25	linear	0.01	0.88	0.92	0.90
		40	41	22	sigmoid	0.1	0.85	0.91	0.88
		50	38	21	sigmoid	0.1	0.87	0.90	0.89
		60	34	19	sigmoid	1	0.85	0.86	0.86
	6	5	57	25	sigmoid	0.1	0.90	0.81	0.86
		10	56	25	radial	3	0.91	0.79	0.85
		20	52	25	linear	0.01	0.89	0.80	0.85
		30	44	25	sigmoid	1	0.86	0.91	0.89
		40	42	22	linear	0.01	0.83	0.86	0.85
		50	38	21	sigmoid	1	0.84	0.86	0.85
		60	34	20	radial	1	0.85	0.90	0.88
Polar bear	24	5	14	37	radial	4	0.79	0.92	0.86
		10	12	27	polynomial	50	0.75	0.95	0.85
		20	9	25	sigmoid	1	0.91	0.78	0.85
		30	8	22	sigmoid	5	0.88	0.81	0.84
		40	6	20	radial	1	0.83	1.00	0.92
		50	5	16	linear	0.01	0.96	0.89	0.93
		60	5	16	polynomial	0.1	0.80	1.00	0.90

In addition to fitting SVM models to the entire dataset (Table 1), we used randomly selected subsets of grizzly bear data (polar bear samples sizes were typically too small to facilitate evaluations involving random subsets of data) to explore how classification accuracy was influenced by: (1) sample size, which we evaluated by holding the post-departure period constant (30 days) and varying the number of bear-years used to train the SVM model (from 10 to 50 in increments of 10, divided equally among classes) and (2) length of the post-departure period, which we evaluated by holding sample size constant (*n*_*present*_ = 15, *n*_*absent*_ = 15) and varying the number of days after den departure used to calculate movement metrics (Table 2). Because classification accuracy can vary according to the subset of samples selected, we estimate median values of classification accuracy, sensitivity, and specificity from 100 iterations of randomly selecting subsets of data comprised of the specified period length and sample size, then performing the leave-two-out cross-validation process described above (Table 2).

**Table 2:** Influence of sample size (*n*; number of adult females in total and those with cubs present or absent) and period length (days since den departure) on the accuracy of identifying cub presence or absence from movement metrics of adult female grizzly bears based on support vector machine models. Sensitivity (proportion of samples with cubs present that were classified correctly), specificity (proportion of samples with cubs absent that were classified correctly), and classification accuracy (proportion of all samples that were classified correctly) are median values from 100 iterations of randomly selecting a subset of data comprised of the specified period and sample size, then performing a leave-two-out cross-validation process that included: (1) withholding two samples (one cub-present and one cub-absent) for testing, (2) training tuned support vector machines with the remaining data, (3) predicting the classification of the two test samples, and then (4) repeating this process for each possible combination of testing and training samples

Analysis	Period	*n*_total_	*n*_present_	*n*_absent_	Sensitivity	Specificity	Accuracy
Sample size	30	10	5	5	0.80	0.70	0.76
	30	20	10	10	0.85	0.83	0.84
	30	30	15	15	0.90	0.88	0.90
	30	40	20	20	0.90	0.90	0.90
	30	50	25	25	0.88	0.92	0.90
Period length	5	30	15	15	0.85	0.80	0.83
	10	30	15	15	0.84	0.79	0.80
	20	30	15	15	0.85	0.80	0.82
	30	30	15	15	0.81	0.87	0.84
	40	30	15	15	0.83	0.86	0.84
	50	30	15	15	0.84	0.86	0.84
	60	30	15	15	0.84	0.84	0.85

Finally, for polar bears, although the only samples for which cubs were known to be absent were from females that did not den, a sample of seven adult females that departed dens in January were available for evaluation. Because den departure in our sample area typically occurs in March and April [8], these samples likely represented failed reproductive events because cubs that depart dens in January are unlikely to have attained the size and strength necessary to survive outside of the den [46]. To test whether SVM models trained with data from non-denning polar bears (representing the cub-absent class) can separate movements of females that denned and departed with cubs from those that departed without cubs, we used the 5-, and 10-, and 20-day SVM models (Table 1; data were not available for all samples for periods > 20 days) to predict presence or absence of cubs among this sample with the premise that all would be classified as cub-absent based on their early den departure dates. Support for this hypothesis would suggest that classification results from the SVM model are based on differences in movements attributable to the presence or absence of cubs and not to differences inherent to denning (e.g., changes in physiology that occur during denning that could manifest in post-departure movements).

## Results

Movement metrics used in SVMs typically differed between females with and without cubs for both species and across post-departure periods but ranges for the two groups overlapped substantially (Fig. 1; Supplementary Table S1). For both species and across periods, mean values for metrics related to distance traveled (i.e., cumulative daily net displacement; number of days with net displacement < 1, >2, and > 5 km for grizzly bears and < 5, >10, and > 25 km for polar bears) indicated less distance traveled by females with cubs relative to those without cubs (Fig. 1; Supplementary Table S1). Mean maximum net-squared displacement was lower for female grizzly bears with cubs relative to those without cubs for all periods, but for polar bears, mean values were lower for females with cubs relative to those without cubs during the first 20 days post-departure but were more similar at longer periods (Supplementary Table S1). Strength of directionality (i.e., high mean resultant lengths representing less tortuous movements) was greater, on average, for polar bears with cubs across periods relative to those without cubs. Strength of directionality for grizzly bears, however, was lower for females with cubs relative to those without cubs only during the first 10 days post-departure (Supplementary Table S1).

Although it was not possible to differentiate females with or without cubs using any single movement metric due to overlap in values (Fig. 1; Supplementary Table S1), SVMs that included all six metrics collectively were able to separate these classes with accuracies exceeding 90% at some of the time periods for both species (Table 1). When tuned SVMs were trained with all available grizzly bear data standardized to 24-hour intervals, accuracy of predicting cub presence or absence was 87% with only 5 days of post-departure data and met or exceeded 90% for 20- or 30-day post-departure periods; accuracy was highest at 92% with 20 days of post-departure data (Table 1). Standardizing locations of grizzly bears to 6-hour intervals did not improve accuracy relative to 24-hour intervals. When SVMs were trained with movement metrics calculated from 6-hour location estimates, accuracy of predicting cub presence or absence was 86% with 5 days of post-departure data and increased to a maximum of 89% with 30 days of data (Table 1). For grizzly bears, sensitivity exceeded or was equal to specificity (i.e., SVMs were more likely to correctly identify cub presence than cub absence) for periods ≤ 20 days but specificity exceeded sensitivity for periods > 20 days (Table 1).

Similar to grizzly bears, accuracy of predicting cub presence or absence for polar bears with tuned SVMs was 86% with only 5 days of post-departure data and exceeded 90% for periods ≥ 40 days post-departure; accuracy was highest at 93% with 50 days of post-departure data (Table 1). Sensitivity did not differ from specificity consistently across periods for polar bears (Table 1). The seven polar bear females that departed dens in January were all classified as cub-absent with the tuned 5-day and 10-day SVM models described in Table 1, 6 of 7 were classified as cub-absent by the 20-day model.

**Fig. 2:**
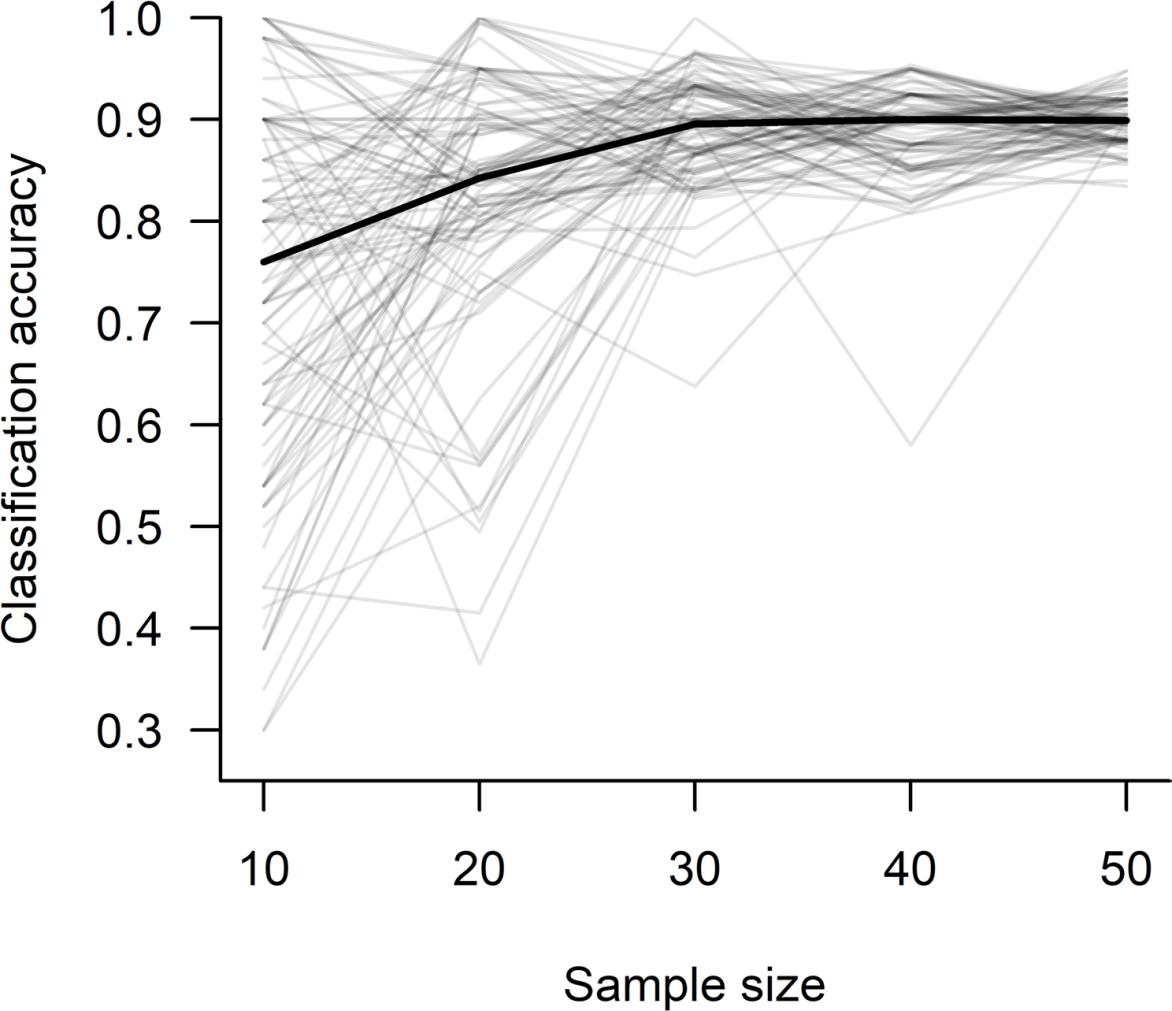
Effect of sample size on accuracy of identifying cub presence or absence based on movement metrics of adult female grizzly bears during the first 30 days after den departure. Training data were split equally between bears with and without cubs. The black line is the median of 100 iterations (gray lines) of randomly selecting a subset of data comprised of the specified sample size, then performing a leave-two-out cross-validation process that included: (1) withholding two samples (one cub-present and one cub-absent) for testing, (2) training tuned support vector machines with the remaining data, (3) predicting the classification of the two test samples, and then (4) repeating this process for each possible combination of testing and training samples

**Fig. 3:**
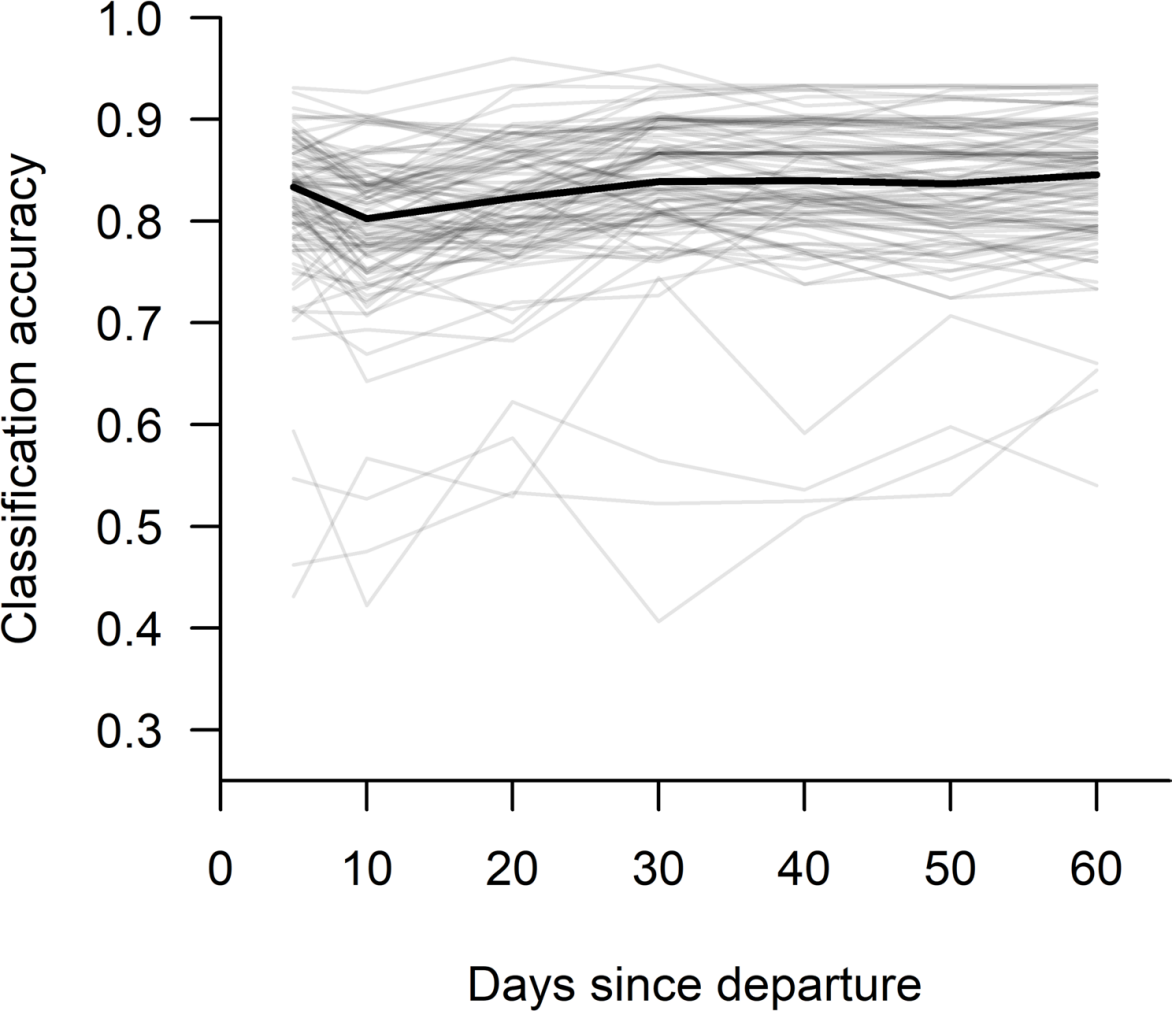
Effect of the length of the post-den departure period on accuracy of identifying cub presence or absence based on movement metrics of adult female grizzly bears. Models included cumulative metrics at day 5- and at 10-day intervals for days 10 to 60 after den departure while the sample size of training data was held constant (*n*_present_ = 15, *n*_absent_ = 15). The black line is the median of 100 iterations (gray lines) of randomly selecting a subset of data comprised of the specified sample size, then for each period, performing a leave-two-out cross-validation process that included: (1) withholding two samples (one cub-present and one cub-absent) for testing, (2) training tuned support vector machines with the remaining data, (3) predicting the classification of the two test samples, and then (4) repeating this process for each possible combination of testing and training samples

For both species, sample sizes decreased as the length of the post-departure period increased, either because collars ceased recording or transmitting data during the 60-day period, or in the case of grizzly bears, the time series was truncated after the final observation that confirmed cub presence or absence. Because this made it difficult to separate the effects of sample size from length of the post-departure period on classification accuracy, we used subsets of grizzly bear data to evaluate the effects of these characteristics. Increasing sample size with training data split equally among cub-present and cub-absent classes increased classification accuracy from 76% at *n* = 10 to 90% at *n* = 30 (medians from 100 iterations of random sampling); accuracy did not increase at sample sizes > 30 (Table 2; Fig. 2). When sample size was held constant (*n*_*present*_ = 13, *n*_*absent*_ = 13), increasing the length of the post-departure period did not affect classification accuracy markedly, with median values ranging from 80 to 85% across periods (Table 2; Fig. 3). Typically, sensitivity exceeded specificity at lower sample sizes and shorter periods when other values were held constant (Table 2).

## Discussion

Our results demonstrated that cub presence or absence can be predicted with high accuracy for both grizzly and polar bears using SVMs trained with post-den departure location data from adult females, even when period lengths and sample sizes were limited. Classification accuracy for both species exceeded 85% with only 5 days of post-departure data and exceeded 90% with 20 (grizzly bear) or 40 (polar bear) days of data (Table 1). Because increases in the length of the post-departure period were always associated with decreases in sample size in our dataset, classification accuracies based on SVMs trained with all available data potentially represented trade-offs between these characteristics. However, our evaluations of the effects of sample size and length of the post-departure period on classification accuracy with subsets of grizzly bear data demonstrated that cub presence or absence can be predicted with accuracies ≥ 90% when SVMs are trained with as few 15 samples from each class with data spanning only the first 30 days after den departure (Table 2; Fig. 2). Further, the lack of substantial increases in accuracy with increases in the length of the post-departure period suggests that this level of accuracy may be possible with < 30 days of post-departure data (Table 2; Fig. 3).

Although our polar bear dataset was too small to permit similar evaluations of the effects of sample size and period length, the fact that classification accuracies > 90% were achieved with as few as 5 cub-present and 16 cub-absent samples (Table 1) suggests that, for some denning species, high accuracy can be achieved with even fewer than 15 samples of each class. In our polar bear dataset, the cub-absent class used to train SVMs consisted of adult females that did not den and, therefore, were known to be without cubs. We acknowledge, however, that bears that emerge from dens in the spring without viable cubs (or those that lose them very quickly) might move differently from bears that did not den due to effects related to the act of denning (e.g., physiological changes due to fasting or long periods of immobility). Consequently, we were interested in knowing whether models trained with data from non-denning bears as the cub-absent class could be used to accurately separate movements of females that denned and departed with cubs from those that departed without cubs. When SVMs trained with all available polar bear data during the 5-, 10-, and 20-day periods (Table 1) were used to classify 7 samples of bears that departed dens in January—when cubs were unlikely to have developed adequately to survive outside the den—all were classified as cub-absent with the 5- and 10-day models and 6 of 7 bears were classified as cub-absent by the 20-day model. This suggests that SVMs trained with data from non-denning bears as the cub-absent class can accurately discriminate between denning bears with and without cubs at den departure, which could be useful for addressing management issues or research questions, either retrospectively or in real-time.

Because our collar location data varied in acquisition rates, location error, and the prevalence of missing data, we used continuous-time correlated random walk models [38, 39] to predict maximum likelihood locations at 24-hour intervals for both species. Although this approach allowed us to standardize location estimates and associated movement metrics used in SVMs across individuals and species, we expected some fine-scale movements that likely differed between classes to be lost. This could be especially important if bears with cubs differed systematically from those without cubs in terms of their movements at scales < 24 h. For example, if movements of bears without cubs were, on average, more tortuous than those with cubs, daily step lengths could be similar for both classes despite bears without cubs traveling greater distances within a day. For this reason, we expected metrics calculated from locations of grizzly bears estimated at 6-hour intervals to result in higher classification accuracy relative to those estimated at 24-hour intervals, but this was not the case. The ability of SVMs to predict cub presence or absence did not differ substantially between SVMs that used movement variables calculated from 6-hour vs. 24-hour location estimates, with highest accuracy slightly lower (3% points) when locations were estimated at 6-hour intervals. This finding suggests that there were likely no important differences in fine-scale movements between classes in our dataset that were not captured by cumulative daily metrics. However, we note that for many classification problems to which SVMs or other machine learning approaches could be applied, it could be beneficial to consider the smallest scale of data applicable to all individuals when choosing variables. If substantial differences in acquisition rates and location error do not exist, for example, the variables used in SVMs could be calculated from collar locations without the step of standardization, possibly capturing nuances in movement important to the species of interest.

We achieved high rates of correctly identifying cub presence or absence with SVMs trained with simple movement metrics calculated from standardized locations that were applicable to both bear species. Although our objective was not to identify variables that would maximize accuracy, we note that including other types of information relevant to the species of interest would likely further increase classification accuracy. For example, variables relating to elevation could be useful in grizzly bear models because in our study area, females with cubs often remain at higher elevations after den departure, possibly to reduce vulnerability of their cubs to mortality by adult male bears that use lower elevations in spring where food is more abundant [14].

Differences in movement metrics between species and cub status generally aligned with our expectations. Polar bears traveled more, on average, than grizzly bears, with cumulative daily net displacement 60 days after den departure being 4.5 times greater for polar bears without cubs and 8.0 times greater for polar bears with cubs relative to corresponding classes of grizzly bears (Supplementary Table S1). For both species, mean cumulative daily net displacement was greater for females without cubs relative to those with cubs. Mean maximum net-squared displacement was always lower for female grizzly bears with cubs relative to those without cubs, whereas for polar bears, values were lower for females with cubs during the first 10 days post-departure but were higher at longer periods (Supplementary Table S1). Similarly, strength of directionality was always greater for polar bears with cubs relative to those without cubs, whereas for grizzly bears, directionality was lower for females with cubs relative to those without cubs only during the first 10 days post-departure (Supplementary Table S1). These discrepancies may be related to differences in denning ecology and dietary breadth between the two species. Polar bears, particularly those that den on land, often travel long distances immediately after departing the den site to reach productive sea ice habitat where their primary prey, ice seals, are available [8, 47]. In contrast, grizzly bears often den in or near their focal home range and their wide dietary breadth means that food can often be found near the den, reducing the need for long movements that could increase their exposure to predators [14].

Because individual bears varied greatly in movement within classes with overlap between cub-present and cub-absent females in all metrics (Fig. 1; Supplementary Table S1), we note that the ability of SVMs to correctly classify cub presence or absence is dependent on the characteristics of the available samples. When sample sizes are especially limited (e.g., for polar bears during periods ≥ 40 days post-departure; Table 1), the inclusion of even one sample with characteristics more typical of the other class could lower accuracy substantially. Thus, although it is possible to achieve high accuracy with very few samples, especially when differences among classes are very pronounced, including data from additional individuals would likely reduce the potential for erroneous inference.

Our finding that classification accuracy did not increase with increasing duration of the post-departure period for grizzly bears (when sample size was held constant; Table 2; Fig. 3) suggests that cub presence or absence can be predicted with collared data within only a few days of den departure (Table 2; Fig. 3). This matched our expectation because cub size and mobility are typically at their lowest levels immediately after leaving the den [14, 48], which would make differences in movement between classes most pronounced early after den departure. As cubs acclimate to conditions outside the den and increase in size and strength, movements of adult females with and without cubs would likely attenuate and become increasingly difficult to separate. Consequently, managers attempting to identify cub presence or absence in real-time would likely benefit from retaining variables calculated with data collected soon after departure in SVMs, even when more contemporary data become available for inclusion.

Our approach can be used to improve estimates of litter survival rates by accounting for the period between when bears emerge from dens and are resighted later in the spring. Additionally, the ability to remotely detect whether cubs are present early after departing the den site can help improve our understanding of factors that affect spring cub survival. For example, previous work [7, 8] demonstrated relationships between polar bear den phenology and short-term litter survival. However, these studies required females to be resighted a short period ( ~ < 60 days) after den site departure to know whether the litter survived or not. Our method can help increase sample size by identifying whether a female likely has cubs during this period without the need for resighting bears. This facilitates opportunities for other investigations related to litter survival, such as how environmental or anthropogenic conditions affect short-term litter survival based solely on movement data. We achieved high accuracy in predicting cub presence or absence from adult location data with SVMs despite limited sample sizes, substantial variation in movement patterns across individuals, and models based on simple movement metrics relevant to two species that differ markedly in movement dynamics. This suggests that SVMs and other similar machine learning techniques are likely useful tools applicable to a wide array of species and ecological investigations, especially those where analyses are complicated by a lack of independence of predictor variables or unrealistic assumptions about the distribution of data [22, 49]. For example, future work could focus on using SVMs to identify the timing of litter loss, which could be useful in a variety of applications, including informing the estimation of impact of human activities on bear populations [50]. Lastly, we found that our approach performed well even with older, lower-resolution data that can be crucial for retrospective analyses, providing researchers with the ability to directly compare historic course-scale data to modern fine-scale data.

## Electronic supplementary material

Below is the link to the electronic supplementary material.

Supplementary Material 1

## Data Availability

The data used in this study are available at: 10.5066/P13KQ2U9 [52].
