# Supplementary material for: Identifying presence or absence of grizzly and polar bear cubs from the movements of adult females with machine learning
Source: Mov Ecol. 2025 Jul 4;13:48. doi: 10.1186/s40462-025-00577-y (PMC12226834; doi:10.1186/s40462-025-00577-y)
Supplement: Supplementary file 1 — Supplementary Material 1 [file 40462_2025_577_MOESM1_ESM.docx]

Supplementary information for:

Identifying presence or absence of grizzly and polar bear cubs from the movements of adult females with machine learning

Erik M. Andersen^1,^*, Justin G. Clapp^2^, Milan A. Vinks^3,4^, Todd C. Atwood^5^, Daniel D. Bjornlie^1^, Cecily M. Costello^3^, David D. Gustine^1,6^, Mark A. Haroldson^7^, Lori L. Roberts^3^, Karyn D. Rode^5^, Frank T. van Manen^7^, Ryan R. Wilson^1^

^1^U.S. Fish and Wildlife Service, Marine Mammals Management, 1011 E Tudor Road, Anchorage, AK 99502, USA

^2^Wyoming Game and Fish Department, Large Carnivore Section, 260 Buena Vista, Lander, WY 82520, USA

^3^Montana Fish, Wildlife and Parks, 490 N Meridian Rd, Kalispell, MT 59901, USA

^4^Montana Cooperative Wildlife Research Unit, Wildlife Biology Program, University of Montana, 205 Natural Sciences Building, Missoula, MT 59812, USA

^5^U.S. Geological Survey, Alaska Science Center, 4210 University Drive, Anchorage, AK 99508, USA

^6^National Park Service, Natural Resource Stewardship and Science, Biological Resources Division-Wildlife Conservation, Anchorage, AK 99502, USA

^7^U.S. Geological Survey, Northern Rocky Mountain Science Center, Interagency Grizzly Bear Study Team, 2327 University Way, Suite 2, Bozeman, MT 59715, USA

*Correspondence: Erik M. Andersen, erik_andersen@fws.gov

Supplementary Table S1. Six model variables calculated at seven periods defined by the number of days since den departure used to identify cub presence or absence from the movements of adult female grizzly bears and polar bears with support vector machine models. Movement metrics included cumulative daily net displacement in km (Cumulative DND), days with daily net displacement <1 km for grizzly bears or <5 km for polar bears (DND <1 or 5), days with daily net displacement >2 km for grizzly bears or >10 km for polar bears (DND > 2 or 10), days with daily net displacement >5 km for grizzly bears or >25 km for polar bears (DND > 5 or 25), maximum net-squared displacement in km (Max NSD), and mean strength of directionality expressed from 0 to 1 (Directionality). For each variable, sample size (*n*), mean, and standard error (SE) are provided for adult females with cubs absent or present. Models also included variables for population (grizzly bears) or subpopulation (polar bears) and den departure date (Julian).

|  |  | Polar bear | |  |  |  |  |  |  | Grizzly bear | |  |  |  |  |  |
| --- | --- | --- | --- | --- | --- | --- | --- | --- | --- | --- | --- | --- | --- | --- | --- | --- |
|  |  | Cubs absent | |  |  | Cubs present | |  |  | Cubs absent | |  |  | Cubs present | |  |
| Variable | Days | *n* | mean | SE |  | *n* | mean | SE |  | *n* | mean | SE |  | *n* | mean | SE |
| Cumulative DND | 5 | 37 | 77.6 | 9.4 |  | 14 | 28.3 | 7.1 |  | 25 | 6.2 | 0.8 |  | 57 | 2.0 | 0.3 |
|  | 10 | 27 | 144.5 | 15.8 |  | 12 | 65.9 | 10.9 |  | 25 | 14.7 | 1.9 |  | 55 | 6.1 | 0.9 |
|  | 20 | 25 | 267.7 | 23.0 |  | 9 | 193.2 | 32.8 |  | 25 | 40.0 | 5.2 |  | 52 | 14.9 | 1.7 |
|  | 30 | 22 | 407.0 | 32.2 |  | 8 | 300.9 | 55.7 |  | 25 | 76.7 | 8.8 |  | 42 | 28.0 | 3.1 |
|  | 40 | 20 | 612.5 | 43.9 |  | 6 | 339.0 | 58.0 |  | 22 | 113.8 | 14.7 |  | 41 | 44.9 | 4.5 |
|  | 50 | 16 | 750.4 | 48.3 |  | 5 | 514.0 | 75.7 |  | 21 | 156.1 | 20.6 |  | 38 | 62.7 | 6.2 |
|  | 60 | 16 | 922.9 | 67.5 |  | 5 | 623.4 | 83.7 |  | 19 | 202.7 | 28.3 |  | 34 | 77.7 | 7.1 |
| DND < 1 or 5 | 5 | 37 | 1.4 | 0.2 |  | 14 | 3.1 | 0.6 |  | 25 | 3.0 | 0.3 |  | 57 | 4.5 | 0.1 |
|  | 10 | 27 | 2.5 | 0.5 |  | 12 | 5.2 | 1.1 |  | 25 | 5.7 | 0.5 |  | 55 | 8.6 | 0.2 |
|  | 20 | 25 | 5.5 | 0.8 |  | 9 | 7.2 | 1.8 |  | 25 | 9.4 | 0.8 |  | 52 | 15.8 | 0.5 |
|  | 30 | 22 | 7.2 | 0.9 |  | 8 | 10.5 | 2.5 |  | 25 | 12.1 | 1.0 |  | 42 | 21.8 | 0.9 |
|  | 40 | 20 | 7.7 | 0.9 |  | 6 | 17.7 | 2.6 |  | 22 | 15.6 | 1.2 |  | 41 | 26.6 | 1.1 |
|  | 50 | 16 | 9.1 | 1.1 |  | 5 | 18.8 | 3.1 |  | 21 | 18.1 | 1.5 |  | 38 | 31.2 | 1.4 |
|  | 60 | 16 | 10.1 | 1.5 |  | 5 | 21.2 | 3.4 |  | 19 | 20.5 | 1.7 |  | 34 | 36.1 | 1.7 |
| DND > 2 or 10 | 5 | 37 | 2.5 | 0.3 |  | 14 | 0.9 | 0.4 |  | 25 | 1.1 | 0.2 |  | 57 | 0.2 | 0.1 |
|  | 10 | 27 | 4.9 | 0.5 |  | 12 | 2.1 | 0.6 |  | 25 | 2.6 | 0.4 |  | 55 | 0.7 | 0.2 |
|  | 20 | 25 | 9.7 | 0.9 |  | 9 | 7.2 | 1.8 |  | 25 | 7.0 | 0.9 |  | 52 | 1.9 | 0.3 |
|  | 30 | 22 | 15.2 | 1.2 |  | 8 | 11.4 | 3.1 |  | 25 | 12.6 | 1.2 |  | 42 | 4.1 | 0.7 |
|  | 40 | 20 | 22.9 | 1.4 |  | 6 | 12.2 | 2.9 |  | 22 | 17.7 | 1.6 |  | 41 | 7.0 | 1.0 |
|  | 50 | 16 | 29.6 | 1.9 |  | 5 | 19.0 | 4.0 |  | 21 | 23.7 | 2.0 |  | 38 | 10.1 | 1.3 |
|  | 60 | 16 | 36.5 | 2.5 |  | 5 | 23.4 | 4.6 |  | 19 | 29.6 | 2.3 |  | 34 | 13.0 | 1.5 |
| DND > 5 or 25 | 5 | 37 | 0.9 | 0.2 |  | 14 | 0.1 | 0.1 |  | 25 | 0.1 | 0.1 |  | 57 | 0.0 | 0.0 |
|  | 10 | 27 | 1.7 | 0.4 |  | 12 | 0.1 | 0.1 |  | 25 | 0.7 | 0.2 |  | 55 | 0.1 | 0.1 |
|  | 20 | 25 | 2.8 | 0.5 |  | 9 | 1.0 | 0.5 |  | 25 | 2.3 | 0.6 |  | 52 | 0.3 | 0.1 |
|  | 30 | 22 | 4.1 | 0.8 |  | 8 | 1.9 | 0.9 |  | 25 | 4.9 | 0.8 |  | 42 | 0.5 | 0.2 |
|  | 40 | 20 | 7.0 | 1.1 |  | 6 | 1.8 | 0.8 |  | 22 | 7.9 | 1.3 |  | 41 | 1.4 | 0.4 |
|  | 50 | 16 | 8.7 | 1.3 |  | 5 | 4.4 | 1.5 |  | 21 | 11.0 | 1.7 |  | 38 | 2.1 | 0.5 |
|  | 60 | 16 | 10.4 | 1.6 |  | 5 | 4.6 | 1.7 |  | 19 | 14.5 | 2.3 |  | 34 | 2.2 | 0.6 |
| Max NSD | 5 | 37 | 4.8×10^6^ | 1.3×10^6^ |  | 14 | 9.3×10^5^ | 4.0×10^5^ |  | 25 | 2.0×10^4^ | 6.0×10^3^ |  | 57 | 4.0×10^3^ | 1.4×10^3^ |
|  | 10 | 27 | 1.2×10^7^ | 3.0×10^6^ |  | 12 | 3.1×10^6^ | 1.1×10^6^ |  | 25 | 1.3×10^5^ | 4.7×10^4^ |  | 55 | 4.0×10^4^ | 2.0×10^4^ |
|  | 20 | 25 | 2.4×10^7^ | 5.6×10^6^ |  | 9 | 2.6×10^7^ | 1.2×10^7^ |  | 25 | 6.9×10^5^ | 4.7×10^5^ |  | 52 | 1.0×10^5^ | 3.1×10^4^ |
|  | 30 | 22 | 2.7×10^7^ | 4.6×10^6^ |  | 8 | 7.5×10^7^ | 3.4×10^7^ |  | 25 | 1.2×10^6^ | 6.0×10^5^ |  | 42 | 1.3×10^5^ | 4.2×10^4^ |
|  | 40 | 20 | 4.5×10^7^ | 8.2×10^6^ |  | 6 | 6.6×10^7^ | 2.6×10^7^ |  | 22 | 1.8×10^6^ | 9.5×10^5^ |  | 41 | 2.2×10^5^ | 6.1×10^4^ |
|  | 50 | 16 | 5.7×10^7^ | 1.1×10^7^ |  | 5 | 1.3×10^8^ | 4.2×10^7^ |  | 21 | 1.9×10^6^ | 1.0×10^6^ |  | 38 | 2.5×10^5^ | 6.2×10^4^ |
|  | 60 | 16 | 9.5×10^7^ | 2.6×10^7^ |  | 5 | 1.5×10^8^ | 4.3×10^7^ |  | 19 | 2.5×10^6^ | 1.3×10^6^ |  | 34 | 2.7×10^5^ | 6.9×10^4^ |
| Directionality | 5 | 37 | 0.62 | 0.04 |  | 14 | 0.81 | 0.06 |  | 25 | 0.54 | 0.04 |  | 57 | 0.44 | 0.03 |
|  | 10 | 27 | 0.43 | 0.05 |  | 12 | 0.71 | 0.07 |  | 25 | 0.37 | 0.04 |  | 55 | 0.31 | 0.03 |
|  | 20 | 25 | 0.29 | 0.03 |  | 9 | 0.59 | 0.08 |  | 25 | 0.23 | 0.03 |  | 52 | 0.23 | 0.02 |
|  | 30 | 22 | 0.24 | 0.03 |  | 8 | 0.63 | 0.08 |  | 25 | 0.15 | 0.02 |  | 42 | 0.16 | 0.02 |
|  | 40 | 20 | 0.20 | 0.03 |  | 6 | 0.58 | 0.07 |  | 22 | 0.12 | 0.02 |  | 41 | 0.15 | 0.02 |
|  | 50 | 16 | 0.18 | 0.03 |  | 5 | 0.53 | 0.09 |  | 21 | 0.11 | 0.01 |  | 38 | 0.14 | 0.02 |
|  | 60 | 16 | 0.18 | 0.02 |  | 5 | 0.50 | 0.06 |  | 19 | 0.10 | 0.01 |  | 34 | 0.12 | 0.02 |
